# Supplementary figures and images for: Identification and characterization of biomarkers associated with endoplasmic reticulum protein processing in cerebral ischemia-reperfusion injury
Source: PeerJ. 2024 Jan 2;12:e16707. doi: 10.7717/peerj.16707 (PMC10768662; doi:10.7717/peerj.16707)

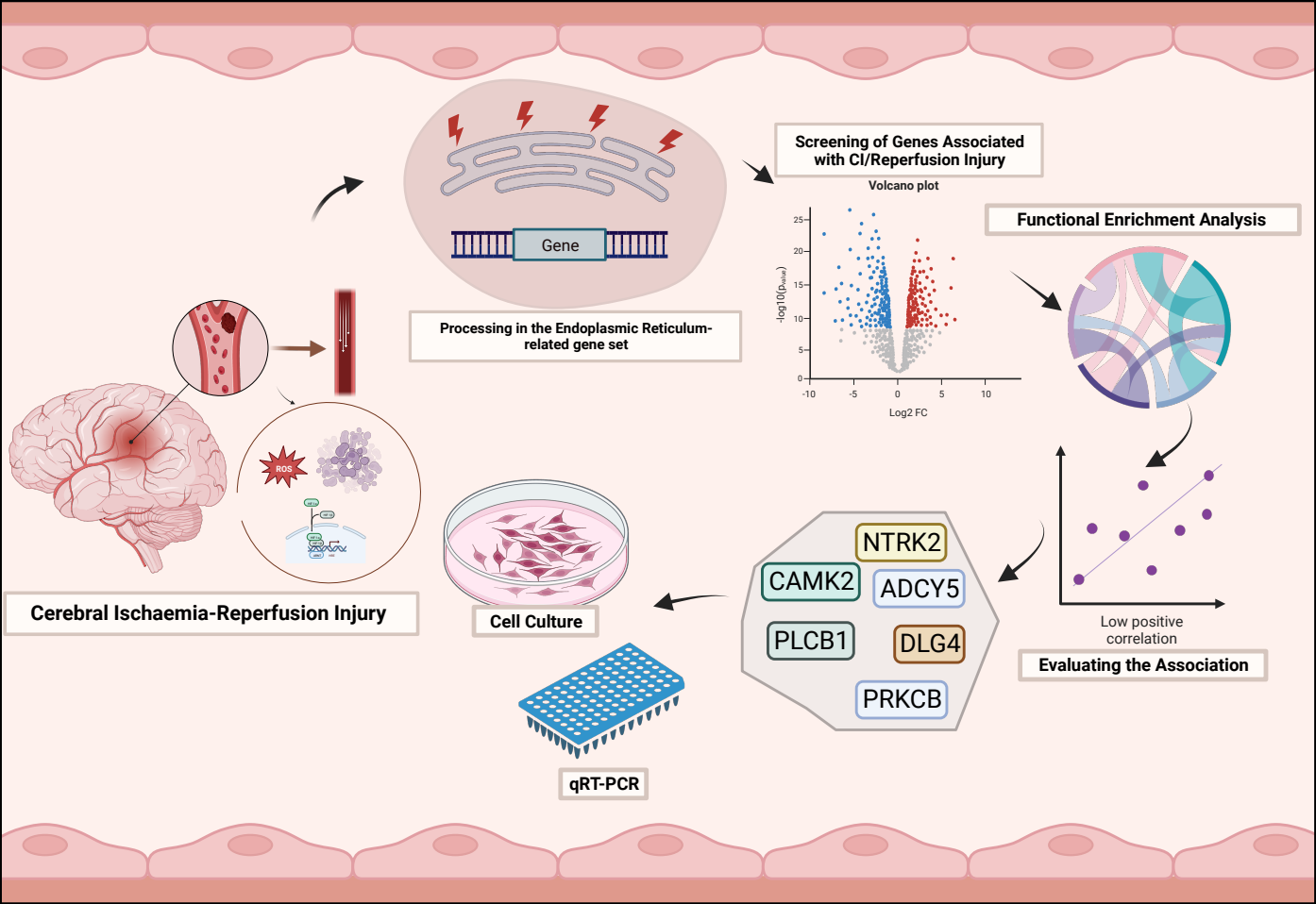

Supplement: Figure S1 [file peerj-12-16707-s001.pdf]
