# Supplementary material for: Identification and characterization of biomarkers associated with endoplasmic reticulum protein processing in cerebral ischemia-reperfusion injury
Source: PeerJ. 2024 Jan 2;12:e16707. doi: 10.7717/peerj.16707 (PMC10768662; doi:10.7717/peerj.16707)
Supplement: Table S1 [file peerj-12-16707-s002.docx]

Supplementary Table 1 The top 10 genes with the largest differences in expression between up- and down-regulation

|  | Differentially express | logFC | AveExpr | t | B | P | adjust P |
| --- | --- | --- | --- | --- | --- | --- | --- |
| *CCL2* | Up | 9.419 | 5.159 | 10.579 | 1.346 | 0.000 | 0.019 |
| *SERPINE1* | Up | 8.297 | 6.190 | 10.936 | 1.502 | 0.000 | 0.018 |
| *CCL7* | Up | 7.721 | 4.136 | 7.579 | -0.246 | 0.001 | 0.030 |
| *IL11* | Up | 7.480 | 4.288 | 9.835 | 1.000 | 0.000 | 0.021 |
| *SPP1* | Up | 7.422 | 7.307 | 17.770 | 3.658 | 0.000 | 0.009 |
| *TIMP1* | Up | 7.182 | 7.381 | 11.553 | 1.759 | 0.000 | 0.017 |
| *LCN2* | Up | 7.077 | 5.548 | 11.135 | 1.587 | 0.000 | 0.017 |
| *CXCL1* | Up | 6.929 | 3.464 | 21.441 | 4.390 | 0.000 | 0.007 |
| *S100A9* | Up | 6.881 | 4.065 | 10.690 | 1.395 | 0.000 | 0.018 |
| *HSPB1* | Up | 6.851 | 7.197 | 12.243 | 2.028 | 0.000 | 0.016 |
| *PLK5* | Down | -3.815 | 2.672 | -6.313 | -1.117 | 0.002 | 0.040 |
| *DDC* | Down | -3.247 | 2.677 | -5.750 | -1.555 | 0.003 | 0.048 |
| *GH1* | Down | -3.151 | 1.576 | -6.153 | -1.238 | 0.002 | 0.042 |
| *BTBD17* | Down | -3.012 | 5.444 | -7.522 | -0.283 | 0.001 | 0.030 |
| *MAMSTR* | Down | -2.858 | 2.115 | -8.418 | 0.256 | 0.001 | 0.028 |
| *NOTUM* | Down | -2.673 | 1.682 | -6.702 | -0.834 | 0.002 | 0.036 |
| *P2RY12* | Down | -2.625 | 5.681 | -12.874 | 2.258 | 0.000 | 0.015 |
| *DIPK1C* | Down | -2.478 | 3.875 | -11.061 | 1.556 | 0.000 | 0.017 |
| *P2RX2* | Down | -2.444 | 1.567 | -5.820 | -1.499 | 0.003 | 0.047 |
| *SCD3* | Down | -2.391 | 1.195 | -6.391 | -1.059 | 0.002 | 0.039 |
