# Supplementary material for: Identification and characterization of biomarkers associated with endoplasmic reticulum protein processing in cerebral ischemia-reperfusion injury
Source: PeerJ. 2024 Jan 2;12:e16707. doi: 10.7717/peerj.16707 (PMC10768662; doi:10.7717/peerj.16707)
Supplement: Table S2 [file peerj-12-16707-s003.docx]

**Table 2 Differentially expressed genes highly correlated with PER score**

| Symbol | Correlation | Symbol | Correlation | Symbol | Correlation | Symbol | Correlation | Symbol | Correlation | Symbol | Correlation | Symbol | Correlation | Symbol | Correlation |
| --- | --- | --- | --- | --- | --- | --- | --- | --- | --- | --- | --- | --- | --- | --- | --- |
| *KCTD11* | 0.964 | *RRBP1* | 0.979 | *CMTM6* | 0.990 | *HSPA4L* | -0.888 | *SEPTIN7* | -0.938 | *SORT1* | -0.953 | *RUSC2* | -0.987 | *SPTB* | -0.989 |
| *PLEK2* | 0.964 | *RBFOX3* | -0.958 | *CHRNA7* | -0.947 | *AI467606* | 0.874 | *FERMT3* | 0.992 | *DDX41* | 0.938 | *PCP4* | -0.895 | *DYNLL2* | -0.970 |
| *ALX1* | 0.964 | *BMP2K* | 0.973 | *BCAN* | -0.941 | *FAM110C* | 0.978 | *C1QB* | 0.991 | *TMEFF1* | -0.931 | *WDR93* | -0.868 | *GRM7* | -0.855 |
| *IL12B* | 0.964 | *RPS3A* | 0.977 | *TGIF1* | 0.989 | *LMNA* | 0.980 | *RAB3IL1* | 0.853 | *ACSL3* | -0.870 | *ESAM* | 0.936 | *CD38* | 0.855 |
| *PABPC4L* | -0.958 | *GBP7* | 0.887 | *SPRY1* | 0.936 | *PLA2G4A* | 0.975 | *ATP2B2* | -0.981 | *AC099450.1* | 0.980 | *EMP1* | 0.930 | *ICAM1* | 0.906 |
| *CHN2* | -0.957 | *GIMAP4* | 0.893 | *SPTBN1* | -0.985 | *RGD1307461* | -0.970 | *RBP1* | 0.993 | *USP1* | 0.956 | *WWC1* | -0.967 | *DNAJB11* | 0.979 |
| *MSMP* | 0.964 | *SHANK2* | -0.951 | *AKAP13* | 0.988 | *SLC1A3* | -0.968 | *EIF2S2* | 0.986 | *D5ERTD579E* | -0.986 | *FOXP1* | -0.920 | *ENC1* | -0.868 |
| *SCT* | 0.964 | *HSPH1* | 0.955 | *LRRTM4* | -0.973 | *MANF* | 0.992 | *CST3* | -0.934 | *ELK3* | 0.979 | *SLC44A4* | 0.950 | *GSAP* | 0.815 |
| *TYRP1* | 0.964 | *BTBD17* | -0.933 | *SOX8* | -0.974 | *ZYX* | 0.967 | *AHCTF1* | 0.902 | *SUSD6* | 0.939 | *MTHFD1L* | 0.994 | *S1PR2* | 0.971 |
| *RPH3A* | -0.967 | *ARHGEF26* | -0.988 | *TP53* | 0.966 | *NABP1* | 0.892 | *S1PR3* | 0.987 | *ATP8B2* | -0.990 | *SIPA1L1* | -0.972 | *CDC42EP4* | -0.904 |
| *ACSL6* | -0.961 | *NOS3* | 0.883 | *HMGB2L1* | 0.883 | *PES1* | 0.959 | *RGS7* | -0.932 | *PFKL* | 0.902 | *TBPL1* | -0.926 | *FNDC1* | -0.889 |
| *PTPRC* | 0.977 | *RASGRP2* | -0.957 | *SEC24D* | 0.971 | *PPP2R1B* | 0.950 | *CARD11* | 0.897 | *SDAD1* | 0.949 | *LRG1* | 0.954 | *SRGAP1* | 0.950 |
| *ANXA7* | 0.959 | *RELA* | 0.949 | *RGS1* | 0.974 | *NRAS* | 0.876 | *VIM* | 0.991 | *CADM4* | -0.915 | *CDK5R2* | -0.978 | *RASSF1* | 0.965 |
| *CSF2RB* | 0.966 | *AKNA* | 0.938 | *MOXD1* | -0.967 | *SLC27A1* | -0.977 | *ITGB2* | 0.978 | *TMEM120A* | 0.977 | *BRSK1* | -0.988 | *PLOD3* | 0.956 |
| *FNDC5* | -0.948 | *RPL10A* | 0.945 | *MYC* | 0.998 | *GABRA1* | -0.978 | *RPSA* | 0.981 | *EPYC* | -0.889 | *EGFL6* | -0.938 | *KCNJ4* | -0.980 |
| *MCOLN3* | 0.916 | *VASP* | 0.962 | *ARHGAP8* | 0.865 | *SLC2A13* | -0.932 | *IL1RN* | 0.980 | *SLC66A3* | 0.937 | *GGTA1* | 0.850 | *USF3* | -0.951 |
| *MMP12* | 0.965 | *ECM1* | 0.926 | *IMPDH2* | 0.998 | *PPP1R15B* | 0.942 | *NECTIN1* | -0.964 | *LOX* | 0.924 | *S100A11* | 0.988 | *SLC30A3* | -0.987 |
| *SPN* | 0.954 | *PTX3* | 0.836 | *ACSBG1* | -0.961 | *DGKB* | -0.983 | *SLFN13* | 0.986 | *PRSS35* | 0.970 | *JAK3* | 0.916 | *PLAUR* | 0.920 |
| *MLPH* | 0.982 | *BAG1* | 0.873 | *ADAMTS1* | 0.989 | *TGFB1* | 0.973 | *TIMP1* | 0.963 | *COL7A1* | 0.878 | *PLAU* | 0.974 | *OMG* | -0.973 |
| *ERBIN* | 0.975 | *SLC8A3* | -0.925 | *MMP19* | 0.996 | *DLG1* | -0.854 | *LCP1* | 0.962 | *RPS25* | 0.887 | *RET* | 0.981 | *SNRPD3* | 0.911 |
| *ZFP316* | -0.948 | *ITPKC* | 0.968 | *NETO1* | -0.958 | *ZFP523* | -0.984 | *TAX1BP3* | 0.991 | *WHRN* | -0.969 | *ITGAX* | 0.842 | *TOP3A* | 0.888 |
| *DENND5A* | -0.967 | *SH3RF1* | -0.914 | *PDYN* | -0.923 | *KRCC1* | 0.901 | *NES* | 0.951 | *C3AR1* | 0.996 | *GRIA3* | -0.968 | *RPL27* | 0.946 |
| *FMNL1* | -0.971 | *SS18* | 0.938 | *PAFAH1B3* | 0.978 | *CSF3R* | 0.933 | *ENPP3* | 0.993 | *DGLUCY* | -0.910 | *PDE1B* | -0.985 | *SKAP2* | 0.973 |
| *GPATCH4* | 0.943 | *FBXO34* | -0.928 | *SEC11A* | 0.976 | *BTBD3* | -0.981 | *ACER2* | 0.950 | *R3HDM2* | -0.876 | *FAM167A* | 0.858 | *IMP4* | 0.944 |
| *CXCL1* | 0.957 | *FREM2* | -0.981 | *DPY19L3* | -0.955 | *RAP1B* | 0.990 | *NSMCE2* | 0.965 | *SEC61G* | 0.982 | *RBFOX1* | -0.982 | *ALDH1A1* | -0.920 |
| *TJP2* | 0.955 | *PYGB* | -0.952 | *DNAJC2* | 0.921 | *SCD3* | -0.855 | *TRIL* | -0.969 | *PPAN* | 0.951 | *HMOX1* | 0.929 | *ASH1L* | -0.929 |
| *ADAMTS7* | 0.986 | *HPCA* | -0.931 | *PALLDL1* | 0.989 | *RND2* | -0.851 | *LYN* | 0.957 | *NMT2* | -0.965 | *TRAPPC1* | -0.943 | *MYBBP1A* | 0.908 |
| *DDX21* | 0.981 | *GMNN* | 0.994 | *URB1* | 0.961 | *IL1B* | 0.922 | *GLS2* | -0.936 | *LCP2* | 0.853 | *ZMYND15* | 0.937 | *SCAP* | -0.987 |
| *SEPTIN2* | 0.960 | *BST1* | 0.996 | *UPP1* | 0.983 | *MAX* | 0.893 | *TRH* | 0.938 | *S100A3* | 0.977 | *ERAP1* | 0.890 | *ZFP382* | -0.922 |
| *LRRFIP1* | 0.987 | *CMTM3* | 0.831 | *RPN2* | 0.930 | *SLCO2B1* | -0.880 | *RPS24* | 0.911 | *PITPNM2* | -0.976 | *GSPT2* | -0.915 | *RRP12* | 0.954 |
| *MGLL* | -0.956 | *SLC26A1* | -0.975 | *CD163* | 0.943 | *NACC2* | -0.972 | *ILDR2* | -0.974 | *CXCL14* | -0.956 | *AK4* | -0.928 | *PCM1* | -0.952 |
| *ART4* | -0.949 | *MCAM* | 0.997 | *NEK6* | 0.886 | *LYSMD2* | -0.904 | *RHOC* | 0.994 | *TYW1* | 0.948 | *ASF1B* | 0.946 | *NDUFB4* | -0.952 |
| *SDS* | -0.949 | *LRRC73* | -0.990 | *SF1* | 0.963 | *CHN1* | -0.984 | *TMBIM1* | 0.964 | *PTGER2* | 0.959 | *IVD* | -0.963 | *PPIC* | 0.979 |
| *KCNQ5* | -0.964 | *TNPO1* | 0.903 | *FOSL1* | 0.920 | *AFG3L1* | 0.922 | *MAST3* | -0.979 | *KCNC3* | -0.873 | *SIPA1* | 0.985 | *ELF4* | 0.960 |
| *ZFHX2* | -0.974 | *DAP* | 0.971 | *LDHB* | -0.935 | *CDC42SE2* | -0.962 | *PC* | -0.943 | *TMEM196* | -0.907 | *MTMR11* | 0.968 | *FGF14* | -0.949 |
| *NAPSA* | 0.991 | *ASB11* | -0.956 | *EFCAB2* | -0.932 | *RASSF4* | 0.905 | *FBXW7* | -0.978 | *PABPC4* | 0.949 | *LRP4* | -0.935 | *MTMR2* | 0.968 |
| *CYP4F18* | 0.917 | *CCDC102A* | 0.918 | *SLC25A29* | -0.935 | *PTPN2* | 0.983 | *ATP10D* | 0.958 | *SASH1* | -0.969 | *NOP56* | 0.980 | *FSTL1* | 0.893 |
| *QRICH2* | 0.917 | *ANXA3* | 0.889 | *COL4A2* | 0.953 | *RGS8* | -0.882 | *PPM1B* | -0.955 | *ERMN* | -0.897 | *RPL22* | 0.871 | *RPL29* | 0.957 |
| *GATAD2A* | 0.978 | *ARID5A* | 0.987 | *APOE* | -0.933 | *TOM1L1* | -0.972 | *CNN3* | 0.957 | *CEP83* | -0.953 | *FAM107A* | -0.966 | *PIAS1* | -0.971 |
| *CAMK4* | -0.958 | *RSAD2* | 0.884 | *OSMR* | 0.928 | *SEC61B* | 0.967 | *E2F5* | 0.983 | *PLCL2* | -0.976 | *APOL3* | 0.976 | *NTSR2* | -0.880 |
| *GPNMB* | 0.939 | *GPX1* | 0.889 | *MT2A* | 0.961 | *TMEM44* | -0.937 | *LCN2* | 0.947 | *PNKD* | -0.922 | *RPL5* | 0.956 | *COL16A1* | 0.907 |
| *HCLS1* | 0.925 | *EMILIN1* | 0.966 | *MYBL2* | 0.831 | *PTRH1* | 0.905 | *A3GALT2* | 0.945 | *LIMD1* | 0.918 | *PSMB9* | 0.881 | *CAMK1D* | -0.871 |
| *SPP1* | 0.881 | *HNRNPF* | 0.994 | *ARPC1B* | 0.981 | *SLC38A3* | -0.946 | *S100A6* | 0.966 | *OSBPL8* | -0.869 | *ANP32B* | 0.985 | *F2RL1* | 0.939 |
| *ALDOC* | -0.970 | *NQO2* | 0.911 | *TRIM47* | 0.931 | *CASP4* | 0.856 | *RAD54L* | 0.941 | *YBX1* | 0.980 | *EHD2* | 0.978 | *ITGB1* | 0.976 |
| *CALCA* | 0.983 | *PICALM* | 0.944 | *SGSM2* | -0.962 | *PLEKHF1* | 0.746 | *S100A10* | 0.976 | *RTN4R* | -0.992 | *OPALIN* | -0.914 | *ZMAT1* | -0.972 |
| *DGKZ* | -0.956 | *0610040J01RIK* | -0.918 | *PSME1* | 0.910 | *MYOF* | 0.891 | *DIPK1C* | -0.967 | *PLCB1* | -0.968 | *SMARCD2* | 0.978 | *CA13* | 0.859 |
| *MVP* | 0.989 | *FXYD5* | 0.963 | *ITGAM* | 0.975 | *VAMP3* | 0.888 | *RIPK1* | 0.955 | *RPL34* | 0.974 | *TRPC3* | -0.957 | *PRRX1* | 0.983 |
| *S100A4* | 0.959 | *STMN1* | -0.971 | *TGM1* | 0.910 | *BMPR1B* | -0.956 | *DNTTIP2* | 0.949 | *CHRNB4* | 0.859 | *FBN1* | 0.959 | *NCF4* | 0.967 |
| *SCG3* | -0.976 | *1700028K03RIK* | -0.866 | *ARHGDIB* | 0.951 | *IL13RA1* | 0.947 | *TNFAIP6* | 0.992 | *LYNX1* | -0.957 | *MYO6* | -0.961 | *FBXL17* | -0.980 |
| *EIF4EBP1* | 0.982 | *PLEC* | 0.982 | *SLA2* | -0.871 | *MMP15* | -0.986 | *KIRREL3* | -0.979 | *TM4SF1* | 0.978 | *ITPR1* | -0.891 | *HIPK3* | -0.958 |
| *FJX1* | -0.968 | *NINJ1* | 0.993 | *SLC7A10* | -0.972 | *CHD7* | 0.985 | *SELP* | 0.890 | *PRKCB* | -0.865 | *PNPLA7* | -0.971 | *CGA* | 0.930 |
| *ADORA2A* | -0.944 | *ALCAM* | -0.884 | *MPP3* | -0.902 | *RUVBL2* | 0.994 | *IRF1* | 0.975 | *HAPLN1* | -0.990 | *LAMC1* | 0.964 | *MPPED1* | -0.988 |
| *HK3* | 0.925 | *CYP1B1* | 0.953 | *AXL* | 0.955 | *PLK5* | -0.941 | *FRMPD1* | 0.981 | *ADAM9* | 0.993 | *PFN1* | 0.974 | *THY1* | -0.950 |
| *FCER1G* | 0.969 | *EFNA1* | 0.988 | *STT3A* | 0.938 | *PLAAT5* | 0.926 | *TAGLN2* | 0.977 | *TSPO* | 0.937 | *RPL23A* | 0.928 | *AGT* | -0.984 |
| *CMBL* | -0.943 | *NUP205* | 0.984 | *ITM2A* | -0.977 | *PCDH20* | -0.974 | *EIF1A* | 0.987 | *CDH15* | -0.977 | *FKBP10* | 0.966 | *KCNG4* | -0.996 |
| *MSR1* | 0.972 | *CKAP2* | 0.866 | *TTC7A* | 0.966 | *NEFL* | -0.886 | *AMPD3* | 0.965 | *ABAT* | -0.965 | *CWF19L2* | -0.926 | *LPAR6* | 0.934 |
| *PRDX1* | 0.987 | *MXI1* | -0.966 | *TEX10* | 0.951 | *SLC3A2* | 0.970 | *SERPINE1* | 0.928 | *MEIS1* | -0.888 | *SGTB* | -0.955 | *IQGAP1* | 0.952 |
| *LITAF* | 0.967 | *XIRP1* | 0.885 | *B2M* | 0.952 | *AABR07054578.1* | -0.952 | *OSBP2* | -0.948 | *CTSB* | 0.980 | *IFI30* | 0.931 | *PFKFB4* | 0.823 |
| *TP53BP2* | -0.937 | *TRIB3* | 0.959 | *SCIMP* | 0.945 | *GAL* | 0.897 | *TTYH1* | -0.985 | *MCF2L* | -0.951 | *TAGLN* | 0.865 | *PDCD11* | 0.975 |
| *RIMS1* | -0.959 | *RGD1559896* | -0.975 | *SERPINB11* | 0.945 | *MMP8* | 0.922 | *USH2A* | -0.933 | *HCN2* | -0.971 | *FCGR1A* | 0.887 | *EPHX4* | -0.981 |
| *MYO19* | 0.990 | *TMEM100* | 0.952 | *FGL2* | 0.971 | *MLIP* | -0.978 | *SIGIRR* | 0.934 | *BSN* | -0.997 | *GNAI3* | 0.979 | *PPP1R21* | -0.920 |
| *WDFY4* | 0.918 | *GPM6B* | -0.916 | *MCM4* | 0.883 | *ILRUN* | -0.915 | *PDE10A* | -0.960 | *ARHGEF4* | -0.958 | *MCM10* | 0.765 | *ARNT2* | -0.990 |
| *RGS9* | -0.939 | *SFXN5* | -0.926 | *SLC1A2* | -0.987 | *LARGE1* | -0.898 | *KCNH7* | -0.944 | *SERTAD1* | 0.984 | *AABR07034648.1* | 0.965 | *OSBPL6* | -0.928 |
| *CLIC1* | 0.997 | *HHATL* | -0.987 | *SALL1* | -0.916 | *MBD2* | 0.947 | *XKR5* | 0.944 | *RPS5* | 0.975 | *RBBP9* | -0.994 | *IL10RB* | 0.966 |
| *RPS27L* | 0.946 | *LILRA5* | 0.945 | *LPP* | 0.905 | *DLG4* | -0.968 | *RPS27A-PS1* | 0.973 | *MKI67* | 0.835 | *CAPG* | 0.842 | *CAD* | 0.961 |
| *MAP4* | 0.957 | *EMP3* | 0.984 | *SLC16A1* | 0.943 | *CDH12* | -0.987 | *ARHGAP5* | -0.964 | *MGST3* | -0.939 | *GPR182* | 0.796 | *FAM102A* | -0.849 |
| *NRGN* | -0.943 | *MLC1* | -0.921 | *GPX8* | 0.926 | *PPP3CA* | -0.967 | *TARS1* | 0.928 | *PTPN12* | 0.991 | *SNRNP48* | 0.905 | *LZTS1* | -0.996 |
| *TMED5* | 0.953 | *SMPD3* | -0.964 | *MYH9* | 0.945 | *ENG* | 0.902 | *S100A9* | 0.958 | *FN1* | 0.929 | *ADM* | 0.896 | *MYO9B* | 0.955 |
| *CD44* | 0.975 | *RALGPS1* | -0.956 | *RAPH1* | -0.946 | *RPL6* | 0.918 | *NDRG2* | -0.972 | *RALGDS* | 0.986 | *SPOCK3* | -0.975 | *KALRN* | -0.989 |
| *CCDC184* | -0.939 | *ID4* | -0.923 | *PPP1R14B* | 0.930 | *IFITM2* | 0.938 | *STON2* | -0.985 | *IL2RG* | 0.907 | *TBX4* | 0.957 | *RPLP1* | 0.970 |
| *ARHGEF2* | -0.958 | *KCNQ3* | -0.920 | *CAST* | 0.977 | *RGD1306746* | -0.974 | *CRELD2* | 0.975 | *NOTUM* | -0.897 | *DTNA* | -0.971 | *NFE2L2* | 0.925 |
| *SYNDIG1* | -0.943 | *PLCE1* | 0.906 | *PLXND1* | -0.966 | *FAM205A* | 0.857 | *EIF3A* | 0.971 | *TALDO1* | 0.978 | *BIRC3* | 0.942 | *P2RX2* | -0.915 |
| *HEATR5A* | 0.983 | *OTULINL* | 0.858 | *AABR07052519.1* | 0.992 | *HES2* | 0.857 | *HCAR2* | 0.914 | *FAM110D* | 0.882 | *PENK* | -0.966 | *FTL1* | 0.983 |
| *CCN6* | -0.949 | *ALYREF* | 0.990 | *NGEF* | -0.979 | *PADI4* | 0.857 | *GCA* | -0.933 | *SALL2* | -0.956 | *SYNE1* | -0.986 | *PRKCA* | -0.948 |
| *MEIG1* | -0.949 | *SERPINH1* | 0.981 | *SECTM1B* | 0.796 | *THBS1* | 0.901 | *ITGAL* | 0.950 | *UNC13A* | -0.909 | *MTMR7* | -0.895 | *TRIM54* | -0.918 |
| *ADHFE1* | -0.973 | *CHD9* | -0.970 | *GM6377* | 0.993 | *CX3CL1* | -0.999 | *CCL2* | 0.927 | *FHL3* | 0.973 | *ARF4* | 0.944 | *PIH1D2* | 0.951 |
| *PDPN* | 0.978 | *PLEK* | 0.821 | *MED21* | 0.936 | *MFNG* | 0.920 | *TUBB6* | 0.985 | *TEX21* | 0.902 | *RPS20* | 0.976 | *AMOTL1* | 0.835 |
| *LGALS3* | 0.998 | *DIS3* | 0.967 | *TMEM123* | 0.970 | *ARHGAP30* | 0.888 | *C1QTNF1* | 0.892 | *MELTF* | -0.951 | *FADS6* | -0.945 | *MMD2* | -0.986 |
| *SLC1A5* | 0.994 | *SEC62* | -0.946 | *GPSM3* | 0.895 | *KCNH4* | -0.891 | *YBX3* | 0.989 | *MSANTD3* | 0.993 | *OTUB2* | -0.965 | *RNH1* | 0.943 |
| *RIPK3* | 0.955 | *BAK1* | 0.972 | *AIFM3* | -0.961 | *CACNA2D2* | -0.969 | *PACSIN3* | 0.971 | *ANKRD23* | -0.867 | *SYNCRIP* | 0.983 | *SNRPEL1* | 0.869 |
| *CCNI* | -0.945 | *EIF3J* | 0.946 | *COL4A1* | 0.944 | *TWF1* | 0.972 | *ASPG* | 0.958 | *JPT2* | 0.984 | *SNX18* | 0.998 | *IQSEC3* | -0.871 |
| *PIP4K2C* | -0.976 | *ARHGEF25* | -0.983 | *NOP14* | 0.989 | *IMPG2* | -0.905 | *TK1* | 0.940 | *U2AF1* | 0.965 | *CD86* | 0.752 | *ST3GAL2* | -0.997 |
| *NEFM* | -0.936 | *TRHDE* | -0.981 | *CTTNBP2NL* | 0.991 | *FBXO30* | 0.978 | *TXNRD1* | 0.992 | *LHPP* | -0.952 | *OLFM1* | -0.991 | *CACNB4* | -0.924 |
| *CLEC5A* | 0.982 | *DNAJC3* | 0.975 | *OAS1K* | 0.970 | *AKR1B10* | 0.747 | *ODC1* | 0.993 | *DDX18* | 0.911 | *CHD1* | 0.997 | *SLC6A11* | -0.978 |
| *IFNAR2* | 0.943 | *N4BP1* | 0.914 | *INTS13* | 0.895 | *MAOA* | 0.911 | *PHACTR1* | -0.939 | *MCF2* | -0.867 | *CAMK2A* | -0.995 | *FAM210B* | -0.867 |
| *MDN1* | 0.941 | *CBARP* | -0.956 | *RIMS2* | -0.971 | *MYO7A* | 0.855 | *BCL3* | 0.938 | *MS4A6A* | 0.896 | *IL1R2* | 0.946 | *CRTAPL1* | 0.871 |
| *LTBR* | 0.943 | *TBCCD1* | 0.847 | *NME6* | 0.890 | *BAZ1A* | 0.976 | *TTLL7* | -0.950 | *STAT4* | 0.858 | *FGF13* | -0.967 | *KCNV1* | -0.955 |
| *SHC3* | -0.939 | *FZD6* | 0.943 | *RUFY4* | 0.988 | *HSPA2* | 0.887 | *ZFAND2A* | 0.912 | *PHLPP1* | -0.875 | *ITGB3* | 0.932 | *SRSF2* | 0.969 |
| *SPTSSB* | -0.958 | *CAMTA2* | -0.984 | *RIN3* | 0.991 | *PYCARD* | 0.922 | *ATP1A2* | -0.975 | *PRODH1* | -0.953 | *TLN1* | 0.972 | *RPL23* | 0.964 |
| *CD14* | 0.984 | *PODXL* | 0.942 | *SPAG9* | -0.972 | *MATK* | -0.979 | *PDE7B* | -0.941 | *CCT4* | 0.938 | *EEF1B2* | 0.935 | *PARP3* | 0.940 |
| *LSP1* | 0.979 | *USP43* | -0.890 | *DRD1* | -0.918 | *BTAF1* | 0.989 | *SOX7* | 0.954 | *MED22* | 0.868 | *TNFSF18* | 0.998 | *TBC1D16* | -0.975 |
| *PUS7* | 0.974 | *HAS1* | 0.928 | *ESM1* | 0.882 | *PKDCC* | 0.866 | *PDE8B* | -0.986 | *GNAI2* | 0.882 | *TRIP6* | 0.882 | *NPPC* | -0.826 |
| *ANO6* | 0.998 | *BID* | 0.912 | *CA14* | -0.941 | *NIBAN2* | 0.963 | *CNN2* | 0.971 | *KCNK2* | -0.914 | *AMOT* | -0.941 | *C1S* | 0.931 |
| *RPL36A* | 0.951 | *SEPTIN8* | -0.991 | *TIGD2* | 0.923 | *IL4R* | 0.912 | *DRAM1* | 0.987 | *PSMB8* | 0.976 | *CARMIL1* | -0.991 | *COQ2* | -0.910 |
| *KNG1* | 0.982 | *SLC2A1* | 0.990 | *HHIP* | -0.902 | *ACSF2* | -0.947 | *PLOD1* | 0.977 | *CHCHD10* | -0.989 | *MFAP3L* | -0.886 | *RMDN1* | -0.969 |
| *SBNO2* | 0.982 | *PLA2G4E* | -0.911 | *ITPKA* | -0.989 | *LACTB* | 0.952 | *IFITM3* | 0.931 | *PLAC8* | 0.973 | *GJA4* | 0.871 | *HSF1* | 0.909 |
| *LPCAT4* | -0.965 | *SH3GLB1* | 0.934 | *MYL12A* | 0.991 | *PLPP3* | -0.951 | *CNTFR* | -0.967 | *SPHK1* | 0.935 | *PFKFB3* | 0.990 | *DDC* | -0.970 |
| *PPP1R1B* | -0.936 | *SYT2* | -0.883 | *RASSF8* | 0.970 | *RPS15* | 0.970 | *NTNG2* | -0.929 | *GPRC5B* | -0.897 | *AGAP2* | -0.932 | *PDLIM7* | 0.981 |
| *CEBPD* | 0.995 | *NIP7* | 0.981 | *SCN4B* | -0.906 | *LRCH1* | 0.990 | *CD276* | 0.934 | *XYLB* | 0.863 | *MAP3K10* | -0.977 | *RBL1* | 0.844 |
| *SYNDIG1L* | -0.925 | *WDR1* | 0.946 | *ACOT11* | -0.916 | *ERI1* | 0.983 | *TMEM43* | 0.944 | *RPL9* | 0.971 | *TGM2* | 0.923 | *IFIT3* | 0.904 |
| *RPS17* | 0.966 | *LSM3* | 0.912 | *MTSS2* | -0.989 | *GH1* | -0.777 | *CAP2* | -0.948 | *RPS4X* | 0.937 | *RPL26* | 0.966 | *HCK* | 0.929 |
| *LINGO1* | -0.932 | *GLI3* | -0.952 | *RPL11* | 0.951 | *KLHL5* | -0.971 | *SLC24A2* | -0.983 | *KCNJ9* | -0.960 | *ANK1* | -0.882 | *STK3* | 0.847 |
| *MICAL2* | -0.918 | *CDH6* | -0.884 | *AABR07008904.1* | 0.900 | *CAPN2* | 0.922 | *S100A8* | 0.963 | *PPRC1* | 0.983 | *DRD2* | -0.912 | *KDSR* | 0.959 |
| *SMAP2* | -0.970 | *TNFSF13* | -0.984 | *CAMKK2* | -0.988 | *TBXAS1* | 0.965 | *ARPP21* | -0.943 | *GJA5* | 0.984 | *LGMN* | 0.966 | *ORC2* | 0.910 |
| *SH3KBP1* | -0.977 | *IRF9* | 0.801 | *CDC42BPG* | -0.960 | *SCART1* | 0.731 | *CCL3* | 0.966 | *ARHGAP6* | -0.934 | *APC* | -0.907 | *CYBA* | 0.989 |
| *MMP9* | 0.971 | *SRSF9* | 0.951 | *MAMSTR* | -0.951 | *DENND2C* | 0.927 | *ARHGAP17* | 0.979 | *UTP4* | 0.955 | *SPI1* | 0.974 | *AC128848.1* | 0.866 |
| *SP140* | 0.961 | *ANXA1* | 0.870 | *TXN1* | 0.990 | *RASD2* | -0.891 | *NECTIN2* | 0.975 | *SLC12A2* | 0.963 | *PDLIM4* | 0.910 | *FEN1* | 0.937 |
| *MYD88* | 0.960 | *MAFF* | 0.955 | *APRT* | 0.954 | *ANKRD34B* | -0.912 | *LRIF1* | -0.937 | *CP* | 0.900 | *CPLX2* | -0.989 | *AABR07063279.1* | 0.828 |
| *MME* | -0.961 | *GBP2* | 0.905 | *ABHD3* | -0.912 | *VIPR2* | -0.881 | *TPM4* | 0.987 | *MSLN* | 0.853 | *COL6A3* | 0.987 | *TNNC2* | -0.838 |
| *SEPTIN5* | -0.939 | *KIFC1* | 0.897 | *ZFAND2B* | -0.936 | *FGFR1* | 0.985 | *CD63* | 0.974 | *TTBK1* | -0.909 | *SHANK1* | -0.974 | *PA2G4* | 0.885 |
| *FLNC* | 0.992 | *FCGR2B* | 0.970 | *PLEKHG2* | 0.987 | *RGS16* | 0.973 | *TFCP2L1* | -0.950 | *MOB3A* | 0.944 | *CLEC10A* | 0.781 | *SYDE1* | 0.910 |
| *AI593442* | -0.951 | *RPL31* | 0.972 | *PPP4R1* | 0.979 | *SGCA* | -0.944 | *BAG3* | 0.991 | *NPAS2* | -0.972 | *GNAO1* | -0.935 | *NAT8L* | -0.959 |
| *P2RY12* | -0.910 | *TACC3* | 0.967 | *SCD2* | -0.926 | *NUP58* | 0.920 | *DLGAP3* | -0.982 | *CD9* | 0.977 | *ANXA2* | 0.913 | *CCDC88C* | 0.962 |
| *ETV5* | -0.938 | *PCYT2* | 0.956 | *FBXW4* | 0.919 | *PDS5A* | 0.987 | *GRIN2A* | -0.973 | *MCEMP1* | 0.749 | *PTN* | -0.924 | *NKIRAS1* | -0.931 |
| *GOLM1* | -0.962 | *LIF* | 0.817 | *ANGPT2* | 0.954 | *RHOJ* | 0.887 | *ZFP385B* | -0.976 | *MAP1A* | -0.985 | *CUX1* | -0.948 | *FAM174B* | -0.951 |
| *NTRK2* | -0.947 | *MYO1C* | 0.957 | *FGR* | 0.978 | *MTHFD2* | 0.952 | *FBXL19* | -0.967 | *NFKB1* | 0.975 | *RAB11FIP4* | -0.968 | *TMEM229B* | -0.920 |
| *STAT3* | 0.974 | *RGS6* | -0.873 | *MARK2* | -0.933 | *TMOD3* | 0.976 | *SLFN2* | 0.982 | *LRRC55* | -0.874 | *SLC50A1* | 0.970 | *CEMIP2* | 0.982 |
| *ADCY5* | -0.957 | *TREM1* | 0.817 | *CD164* | 0.980 | *RHOBTB2* | -0.978 | *IL11* | 0.821 | *GABRG1* | -0.984 | *MACROD1* | -0.895 | *PARD6A* | -0.872 |
| *DIXDC1* | -0.920 | *NDNF* | -0.892 | *KRT2* | -0.896 | *PAK1IP1* | 0.976 | *LYRM9* | -0.971 | *FBLN2* | 0.850 | *GFAP* | 0.808 | *KIF1A* | -0.977 |
| *SH3PXD2B* | 0.969 | *SCP2* | -0.952 | *TNFRSF12A* | 0.973 | *GSTK1* | -0.883 | *EPB41L3* | -0.962 | *RAC2* | 0.933 | *ANGPTL4* | 0.878 | *MAP3K6* | 0.880 |
| *TTL* | -0.966 | *CDO1* | 0.979 | *PPFIA3* | -0.954 | *PIP5K1B* | -0.918 | *NMNAT1* | 0.941 | *ZFP462* | -0.950 | *SEC23B* | 0.993 | *PLPPR5* | -0.996 |
| *DSE* | 0.976 | *CDT1* | 0.949 | *FBL* | 0.967 | *MT1* | 0.925 | *CCL4* | 0.870 | *SLC6A13* | -0.905 | *DOCK8* | 0.888 | *PRICKLE1* | -0.867 |
| *MN1* | -0.972 | *PLIN2* | 0.872 | *RRAS* | 0.959 | *NFKB2* | 0.959 | *DUSP16* | 0.971 | *PKIB* | -0.839 | *RPS27A* | 0.929 | *CDK6* | 0.817 |
| *STARD8* | -0.986 | *ALDH1L2* | 0.956 | *MAGI2* | -0.948 | *ABCA2* | -0.915 | *MIDEAS* | 0.975 | *OASL* | 0.962 | *NOP58* | 0.981 | *HNMT* | -0.925 |
| *SLC25A18* | -0.941 | *RPL28* | 0.970 | *PDLIM1* | 0.940 | *EIF3M* | 0.914 | *MCMBP* | 0.940 | *CYP4F1* | -0.860 | *MPC1* | -0.923 | *RBM3* | 0.847 |
| *SLPI* | 0.934 | *APPL2* | -0.912 | *ADAMTS9* | 0.924 | *PVR* | 0.930 | *IDH2* | -0.933 | *SRPK2* | -0.870 | *EPS15* | -0.962 | *NR3C1* | -0.978 |
| *RPS12* | 0.970 | *SAT2* | -0.978 | *PAK1* | -0.959 | *NLGN2* | -0.967 | *PTBP1* | 0.992 | *FNDC3B* | 0.948 | *HSPB8* | 0.984 | *FNIP2* | 0.992 |
| *MARK3* | 0.984 | *IL6R* | 0.916 | *SEMA5A* | -0.913 | *MCM2* | 0.914 | *NPM1* | 0.961 | *CLDN10* | -0.991 | *RPLP0* | 0.971 | *MINK1* | -0.966 |
| *TNFRSF1A* | 0.992 | *SHMT2* | 0.973 | *SLC22A6* | -0.966 | *WDHD1* | 0.929 | *ATF7* | 0.982 | *SLC6A1* | -0.995 | *GLIPR1* | 0.985 | *VSNL1* | -0.965 |
| *MSN* | 0.993 | *LBP* | 0.782 | *ETL4* | -0.937 | *PAWR* | 0.966 | *C1QC* | 0.993 | *FGF12* | -0.892 | *PARPBP* | 0.739 | *GAL3ST3* | -0.964 |
| *HSPB1* | 0.887 | *BCL2A1* | 0.948 | *HS3ST5* | -0.892 | *DPAGT1* | 0.843 | *CNTN4* | -0.912 | *TYROBP* | 0.978 | *FEZ1* | -0.903 | *COQ8B* | 0.947 |
| *STK40* | 0.948 | *PADI2* | -0.951 | *CTSZ* | 0.978 | *ACO2* | -0.942 | *CXCL16* | 0.934 | *CHUK* | 0.947 | *CALU* | 0.966 | *MAPKAPK3* | 0.977 |
| *RORB* | -0.937 | *CRACDL* | -0.987 | *SLC25A24* | 0.950 | *ADRB1* | -0.910 | *SNPH* | -0.967 | *GSTM1* | -0.875 | *ZDHHC8* | -0.987 | *LUZP2* | -0.898 |
| *ATP13A4* | -0.922 | *FAXC* | -0.915 | *GRIP2* | -0.909 | *MYO1F* | 0.949 | *ZFP672* | 0.935 | *OLFM3* | -0.906 | *RPS27* | 0.948 | *ARL3* | -0.889 |
| *IFI27L2B* | 0.961 | *LRR1* | 0.752 | *GRN* | 0.985 | *TTLL1* | -0.923 | *GLIPR2* | 0.817 | *FMN1* | -0.922 | *OLFML2B* | 0.947 | *MIB1* | -0.967 |
| *CARHSP1* | 0.957 | *NETO2* | -0.932 | *CCL7* | 0.895 | *PKN2* | 0.957 | *PLPP1* | 0.943 | *PSMA6* | 0.943 | *IFT80* | -0.938 | *DAGLB* | -0.973 |
| *CADPS2* | -0.983 | *MGC105649* | 0.953 | *ABTB1* | -0.929 | *CHSY1* | 0.960 | *GNL3* | 0.928 | *SPATA5* | 0.974 |  |  |  |  |
